# The predictive effect of direct-indirect bilirubin ratio on clinical events in acute coronary syndrome: results from an observational cohort study in north China

**DOI:** 10.1186/s12872-022-02894-1

**Published:** 2022-11-10

**Authors:** Jiayu Li, Yanguo Xin, Jingye Li, Meng Meng, Li Zhou, Hui Qiu, Hui Chen, Hongwei Li

**Affiliations:** 1 Department of Cardiology, Beijing Friendship Hospital, Capital Medical University, No. 95 Yong’an Road, Xicheng District, 100050 Beijing, China; 2 Department of Internal Medical, Medical Health Center, Beijing Friendship Hospital, Capital Medical University, Beijing, China; 3 Beijing Key Laboratory of Metabolic Disorder Related Cardiovascular Disease, Beijing, China

**Keywords:** Bilirubin, Chinese, Extremely high-risk, ASCVD, DIBIL ratio

## Abstract

**Background:**

Patients with extremely high-risk ASCVD usually suffered poor prognosis, bilirubin is considered closely related to cardiovascular outcomes. However, there is controversy over the relationship between bilirubin and coronary artery disease. This study aimed to evaluate the predictive value of the DIBIL ratio in patients with extremely high-risk ASCVD.

**Methods:**

10,260 consecutive patients with extremely high-risk ASCVD were enrolled in this study. All patients were divided into three groups according to their DIBIL ratio. The incidence of MACCEs was recorded, and in a competing risk regression, the incidence of MACCEs and their subgroups were recorded. The direct-indirect bilirubin ratio (DIBIL ratio) was calculated by the direct bilirubin (umol/L)/indirect bilirubin (umol/L) ratio, all laboratory values were obtained from the first fasting blood samples during hospitalization.

**Results:**

The area under the ROC curve of the DIBIL ratio to predict the occurrence of all-cause death was 0.668, the cut-off value of which is 0.275. Competing risk regression indicated that DIBIL ratio was positively correlated with all-cause death [1.829 (1.405–2.381), p < 0.001], CV death [1.600 (1.103, 2.321), p = 0.013]. The addition of DIBIL ratio to a baseline risk model had an incremental effect on the predictive value for all-cause death [IDI 0.004(0, 0.010), p < 0.001; C-index 0.805(0.783–0.827), p < 0.001].

**Conclusion:**

The DIBIL ratio was an excellent tool to predict poor prognosis, suggesting that this index may be developed as a biomarker for risk stratification and prognosis in extremely ASCVD patients.

**Supplementary information:**

The online version contains supplementary material available at 10.1186/s12872-022-02894-1.

## Introduction

CAD has been the first killer both in China and worldwide [1–3]. According to a report in 2013, the number of CAD deaths had reached 3.72 million in China [2]. For decades, various tools are developed to evaluate the risk of CAD risks, such as the SCORE model in Europe [4], and PCE for ASCVD [5]. According to China’s cardiovascular prevention guideline in 2017, the risk evaluation of ASCVD is necessary to help physicians guide the best preventive approaches via a more accurate estimation of the risk of ASCVD. Patients diagnosed with extremely high-risk ASCVD are associated with a significantly elevated risk of recurrent MACCEs, indicating that early biomarkers or more details of this group of patients may contribute to a positive prognosis.

A large body of evidence reported that many clinical and laboratory factors were associated with the prognosis in ACS patients [6–8], bilirubin is the end-product of heme degradation, presenting in two forms: DB and IDB. IDB could be converted to DB in hepatocytes and excreted into bile acid [9]. Earlier studies reported that bilirubin is a waste product, however, recent evidence indicated that bilirubin possessed protective effects [10]. Animal models of atherosclerosis and myocardial infarction also showed that bilirubin could improve vascular dysfunction. The reported underlying mechanisms included anti-oxidative, anti-inflammatory, and anti-adipogenic effects of bilirubin. However, studies of the association between bilirubin levels and prognosis in CVD patients provided conflicting results, indicating an inverse relationship between bilirubin and mortality [11–14]. After analysis of the characteristics of patients enrolled in these studies, several factors such as the sample size, and the levels of bilirubin may contribute to different even opposite conclusions. In addition, there is still controversy over which parameters (direct bilirubin, indirect bilirubin, total bilirubin, or the ratio) are better to predict the prognosis in ASCVD.

Therefore, this study aims to evaluate whether the direct-indirect bilirubin ratio (DIBIL ratio) at admission could indicate the long-term prognosis of extremely high-risk ASCVD patients in north China.

## Materials and methods

### Study population

**Fig. 1:**
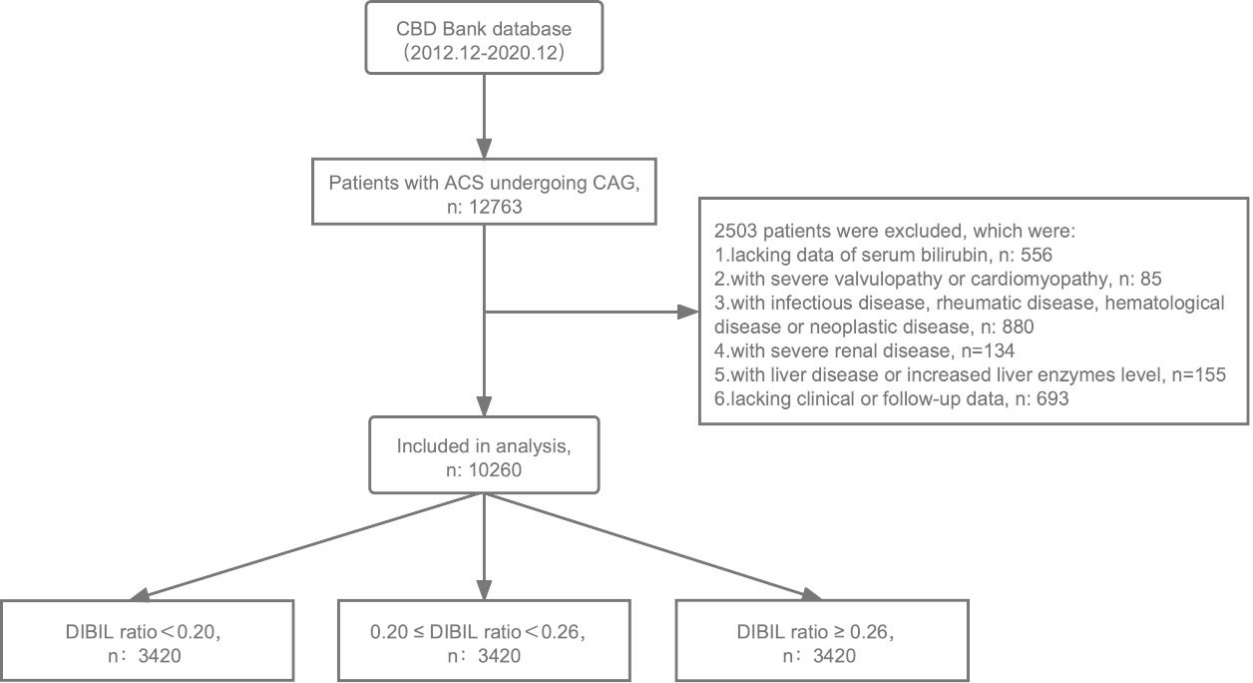
Flow chart of study subject enrollment. (CBD, Cardiovascular Center of Beijing Friendship Hospital Database; ACS, acute coronary syndrome; CAG, coronary angiography; DIBIL ratio, the ratio of direct bilirubin (umol/L)/Indirect bilirubin (umol/L))

All enrolled patients were identified from the Cardiovascular Center of Beijing Friendship Hospital Database (CBD Bank). From Dec 2012 to Dec 2020, 12,763 ACS patients were evaluated as extremely high-risk. According to the 2018 AHA/ACC cholesterol guideline and Consensus of Chinese experts on lipid management in extremely high-risk ASCVD patients, the extremely high-risk ASCVD was identified as those who suffered more than 2 times severe ASCVD events, or those with 1-time severe ASCVD combined with more than 2 high-risk factors (**Supplementary material **1). According to the flow chart (Fig. 1), 2503 were excluded according to the exclusion criteria, including(1) 556 patients lack data of serum bilirubin; (2) 85 patients were diagnosed with severe valvular diseases or cardiomyopathy; (3) 880 patients were meanwhile suffering infectious disease, rheumatic disease, hematological disease or neoplastic disease; (4) 134 patients were diagnosed with severe renal disease; (5) 155 patients with liver disease or increased liver enzymes; (6) 693 patients lost clinical or follow-up data. The final 10,260 included patients were divided into tertiles according to their DIBIL ratio levels (DIBIL ratio < 0.20 group, n = 3420; 0.20 ≤ DIBIL ratio < 0.26 group, n = 3420; DIBIL ratio ≥ 0.26, n = 3420). All patients were followed up till Oct 31, 2021, with a median follow-up of 41.7 months.

### Data collection and definitions

This study was approved by the Institutional Review Board of Beijing Friendship Hospital Affiliated to Capital Medical University and all steps were carried out according to the Declaration of Helsinki. Patients’ basic characteristics, including their medical history, laboratory test values, imaging findings, and angiographic evaluation results were collected and verified by the medical recording system in Beijing Friendship Hospital. All the fasting blood samples were taken on the morning after PCI and the TB and DB and other laboratory parameters were measured by standard methods (the reference range for TB in our hospital is 3.42–17.1 umol/L, 0-6.84 umol/L for DB, and 0–12 umol/L for Indirect bilirubin). The incidence of MACCEs was reported during the hospitalization and follow-up period after the discharge, which was performed with a phone interview.

Clinical comorbidities are defined according to the following criteria: Hypertension: blood pressure ≥ 140/90mmHg three times on at least two days, patients who are receiving antihypertensive drugs. DM: patients meet one of the following criteria: (1) receiving antidiabetic agents; (2) the typical symptoms of DM with FPG ≥ 7.0 mmol/L, and/or RBG ≥ 11.1 mmol/L, and/or 2-h plasma glucose level after OGTT ≥ 11.1 mmol/L. Dyslipidemia: fasting TC > 200 mg/dL, and/or LDL-C > 130 mg/dL, and/or TGs > 150 mg/dL, and/or HDL-C < 40 mg/dL, and/or receiving lipid-lowering drugs. AMI (including NSTEMI and STEMI): chest pain with new ST-segment changes and elevation of myocardial necrosis markers to at least twice the upper limit of the normal range. ACS: acute coronary syndrome (ACS) refers to a group of conditions that include ST-elevation myocardial infarction (STEMI), non-ST elevation myocardial infarction (NSTEMI), and unstable angina.

In this study, MACCEs were defined as all-cause death, CV death, non-fatal MI, stroke, cardiac rehospitalization, or revascularization [15]. CV death was defined as fatal stroke or MI, sudden death. All-cause death was defined as the incidence of death regardless of the reasons. Non-fatal stroke (both ischemic and hemorrhagic stroke) was defined as cerebral dysfunction due to a cerebral vascular occlusion or sudden rupture, which was diagnosed according to the signs of neurological dysfunction or imaging evidence. Cardiac rehospitalization refers to rehospitalization due to angina or heart failure. Any coronary revascularization was defined as revascularization of the target vessel or non-target vessels.

### Statistical analysis

Continuous variables were shown as mean ± standard deviation (SD) or continuous variables with abnormal distribution were expressed as median (25th-75th percentile). Anova or Kruskal Wallis test was applied to compare the difference between groups. Categorical data were illustrated as numbers and percentages. The Pearson chi-square test or Fisher’s exact test was adopted to analyze the difference. Receiver-operating characteristic (ROC) curve analysis was adopted to identify the predictive effect of different markers and their optimal cut-off point value on MACCEs. Basic factors that correlated with all-cause death in the univariate analyzed model were enrolled in the multivariate model. Considering the competitive risk between all-cause death and other outcomes, we imported the competing risk model to identify the independent predictive effect of the DIBIL ratio on the sub-group of MACCEs. Competing risk regression curves were used to estimate the incidence of MACCEs and their subgroups. Integrated discrimination improvement (IDI) was also involved to determine the extent to which the addition of the DIBIL ratio improves the predictive power of the existing baseline risk model. All statistical tests were performed with IBM SPSS statistics 26, Stata/SE 15.1, and the R Programming Language. A two-tailed p-value < 0.05 was regarded as statistically significant.

## Results

### Baseline characteristics of patients

**Fig. 2:**
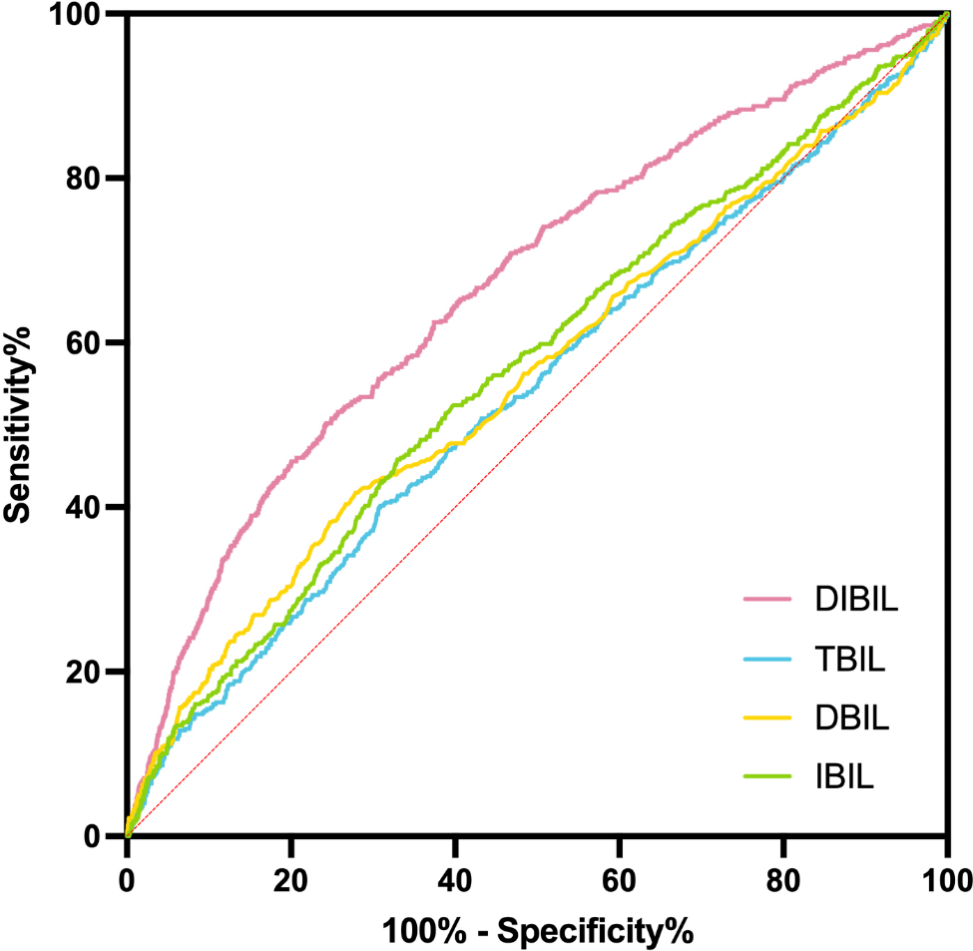
The receiver operating characteristic (ROC) curves of the DIBIL ratio, DBIL, TBIL, and IBIL as markers to predict all-cause death in patients with ACS. The area under ROC curves (AUCs) of the DIBIL for predicting the occurrence of all-cause death was 0.668 (95% CI 0.643–0.694; p < 0.001). The cut-off value of the DIBIL ratio to predict all-cause death was 0.275, the sensitivity was 51.61% and the specificity was 74.29%. (ROC, Receiver-operating characteristic; DIBIL ratio, direct-indirect bilirubin ratio; DBIL, direct bilirubin; TBIL, total bilirubin; IBIL, indirect bilirubin; ACS, acute coronary syndrome)

We finally enrolled 10,260 diagnosed with ACS according to our exclusive and inclusive criteria (Fig. 1). We firstly compared the DIBIL ratio, DBIL, TBIL, and IBIL, and identified that the DIBIL ratio is the best biomarker to predict the all-cause death in our enrolled patients (Fig. 2), the area under ROC curves (AUCs) of the DIBIL ratio for predicting the occurrence of all-cause death was 0.668, the sensitivity was 51.61% and the specificity was 74.29%. Contrasted with DBIL, IBIL and TBIL, DIBIL ratio shows a larger AUC (p < 0.001) (**Supplementary material **2).

All enrolled patients were divided into tertiles according to their DIBIL ratio levels (DIBIL ratio < 0.20 group, n = 3420; 0.20 ≤ DIBIL ratio < 0.26 group, n = 3420; DIBIL ratio ≥ 0.26, n = 3420). Tables 1 and 2 illustrated the baseline and procedural characteristics of all 10,260 patients with complete follow-up information, with available outcomes information.

**Table 1:** Baseline characteristics of the study population

Variable		Total population	Low DIBIL ratio	Moderate DIBIL ratio	High DIBIL ratio	***p*** value
		n = 10,260	n = 3420	n = 3420	n = 3420	
Total bilirubin, umol/L		13.75 ± 6.20	14.17 ± 6.39	13.69 ± 5.78	13.39 ± 6.40	<0.001
Direct bilirubin, umol/L		2.64 ± 1.54	1.97 ± 0.94	2.54 ± 1.08	3.40 ± 2.01	<0.001
Indirect bilirubin, umol/L		11.11 ± 5.06	12.20 ± 5.52	11.15 ± 4.72	9.99 ± 4.67	<0.001
Direct/indirect bilirubin ratio		0.23 (0.18, 0.28)	0.17 (0.15, 0.18)	0.23 (0.21, 0.24)	0.31 (0.28, 0.37)	<0.001
Age, years		63.9 ± 10.3	62.7 ± 10.3	64.1 ± 10.1	65.0 ± 10.4	<0.001
Male gender		6911 (67.4)	1974 (57.7)	2328 (68.1)	2609 (76.3)	<0.001
BMI, kg/m^2^		25.9 ± 3.5	25.8 ± 3.4	25.9 ± 3.5	25.9 ± 3.5	0.386
SBP, mmHg		130.9 ± 18.7	131.8 ± 18.7	131.1 ± 18.6	130.0 ± 18.8	0.001
DBP, mmHg		75.7 ± 11.6	76.3 ± 12.0	75.5 ± 11.5	75.3 ± 11.4	0.001
Heart rate, bpm		72 ± 12	71 ± 12	71 ± 12	72 ± 13	0.261
**Medical history**						
	Current/ex-Smoker	5930 (57.8)	1754 (51.3)	2044 (59.8)	2132 (62.3)	<0.001
	Hypertension	7154 (69.7)	2335 (68.3)	2407 (70.4)	2412 (70.5)	0.043
	Diabetes	3665 (35.7)	1201 (35.1)	1213 (35.5)	1251 (36.6)	0.207
	Dyslipidemia	4897 (47.7)	1695 (49.6)	1689 (49.4)	1513 (44.2)	<0.001
	Previous Stroke	1557 (15.2)	450 (13.2)	531 (15.5)	576 (16.8)	<0.001
	Previous MI	1011 (9.9)	241 (7.0)	344 (10.1)	426 (12.5)	<0.001
	Past PCI	1514 (14.8)	349 (10.2)	530 (15.5)	635 (18.6)	<0.001
	Past CABG	200 (1.9)	46 (1.3)	61 (1.8)	93 (2.7)	<0.001
**Clinical presentation**						
	STEMI	1732 (16.9)	586 (33.8)	531 (30.7)	615 (35.5)	0.700
	NSTEMI	1599 (15.6)	547 (34.2)	527 (33.0)	525 (32.8)	
	UAP	6929 (67.5)	2287 (33.0)	2362 (34.1)	2280 (32.9)	
**Medication on admission**						
	Antiplatelet agent	3790 (36.9)	1075 (31.4)	1331 (38.9)	1384 (40.5)	<0.001
	ACEI/ARB	3502 (34.1)	1114 (32.6)	1208 (35.3)	1180 (34.5)	0.048
	Beta-blocker	2261 (22.0)	691 (20.2)	802 (23.5)	768 (22.5)	0.004
	Statins	3266 (31.8)	975 (28.5)	1204 (35.2)	1087 (31.8)	0.004
**Medication during hospitalization**						
	Antiplatelet agent	9936 (96.8)	3322 (97.1)	3309 (96.8)	3305 (96.6)	0.240
	ACEI/ARB	5706 (55.6)	1848 (54.0)	1863 (54.5)	1995 (58.3)	<0.001
	Beta-blocker	7124 (69.4)	2371 (69.3)	2332 (68.2)	2421 (70.8)	0.189
	Statins	9451 (92.1)	3175 (92.8)	3131 (91.5)	3145 (92.0)	0.178
**Laboratory data**						
	WBC, 10^9^/L	6.7 (5.5, 8.2)	6.8 (5.61, 8.31)	6.6 (5.50, 8.16)	6.7 (5.40, 8.17)	0.006
	Hemoglobin, g/L	135.7 ± 18.6	136.1 ± 18.6	135.8 ± 18.8	135.1 ± 18.3	0.087
	HsCRP, mg/L	1.94 (0.75, 6.06)	1.94 (0.74, 5.72)	1.76 (0.70, 5.28)	2.12 (0.81, 7.35)	<0.001
	RBG at admission, mmol/L	7.4 (6.0, 9.8)	7.4 (5.9, 9.9)	7.3 (6.0, 9.7)	7.4 (6.0, 9.8)	0.877
	FPG, mmol/L	5.5 (4.8, 6.7)	5.5 (4.9, 6.9)	5.4 (4.8, 6.7)	5.4 (4.7, 6.6)	0.002
	HbA1c, %	6.1 (5.6, 7.1)	6.1 (5.6, 7.2)	6.0 (5.6, 7.0)	6.1 (5.6, 7.1)	0.155
	Albumin, g/L	39.0 (36.8, 41.5)	39.7 (37.4, 42.3)	38.9 (36.9, 41.4)	38.4 (36.0, 40.7)	<0.001
	ALT, U/L	19.0 (13.0, 28.0)	18.0 (13.0, 27.0)	19.0 (13.0, 28.0)	19.0 (14.0, 29.0)	<0.001
	AST, U/L	19.8 (16.0, 19.0)	19.0 (15.0, 27.8)	19.7 (16.0, 28.0)	20.0 (16.0, 31.0)	<0.001
	ALP, U/L	75.0 (63.0, 89.0)	76.0 (64.0, 90.0)	76.0 (63.0, 90.0)	74.0 (62.0, 88.0)	0.001
	GGT, U/L	24.0 (17.0, 36.0)	23.0 (17.0, 34.0)	24.0 (17.0, 36.0)	25.0 (17.0, 39.0)	0.002
	ChE,	8.2 (7.2, 9.2)	8.6 (7.7, 9.7)	8.2 (7.3, 9.2)	7.7 (6.7, 8.7)	<0.001
	Creatinine, umol/L	77.0 (66.7, 88.4)	73.3 (62.8, 84.6)	76.9 (67.1, 88.0)	80.3 (70.4, 91.8)	<0.001
	eGFR, ml/min/1.73m2	86.5 (72.9, 99.1)	89.5 (75.7, 101.5)	86.5 (73.0, 99.1)	83.9 (70.2, 96.3)	<0.001
	TC, mmol/L	4.18 (3.53, 4.89)	4.70 (4.06, 5.44)	4.11 (3.53, 4.74)	3.75 (3.18, 4.41)	<0.001
	TGs, mmol/L	1.39 (1.03, 1.98)	1.67 (1.19, 2.38)	1.36 (1.01, 1.86)	1.21 (0.91, 1.70)	<0.001
	LDLC, mmol/L	2.36 (1.89, 2.89)	2.69 (2.20, 3.24)	2.32 (1.89, 2.79)	2.08 (1.68, 2.57)	<0.001
	HDLC, mmol/L	1.04 (0.90, 1.23)	1.09 (0.94, 1.29)	1.03 (0.90, 1.21)	1.00 (0.86, 1.17)	<0.001
**Echocardiography**						
	LVEF	64.5 ± 8.0	64.5 ± 8.0	64.1 ± 8.5	62.8 ± 9.7	<0.001

ACEI: angiotensin-converting enzyme inhibitors, ARB: angiotensin receptor blockers, ALT: alanine aminotransferase, AST: aspartate amino transferase, ALP: alkaline phosphatase, BMI: body mass index, CABG: Coronary Artery Bypass Grafting, CRP: c-reactive protein, ChE: Cholinesterase, DBP: diastolic blood pressure, eGFR: estimated glomerular filtration rate, FPG: fast plasma glucose, GGT: gamma glutamyl transpeptidase, HDL-C: high density lipoprotein cholesterol, LDL-C: low density lipoprotein cholesterol, LVEF: left ventricular ejection fraction, MI: myocardial infarction, NSTEMI: non-ST elevated myocardial infarction, PCI: percutaneous coronary intervention, RBG: random blood glucose, SBP: systolic blood pressure, STEMI:ST-elevated myocardial infarction, TC: total cholesterol, TGs: triacylglycerol, UAP: unstable angina pectoris, WBC: white blood cells

**Table 2:** Angiography characteristics and treatment

Variable		Total population	Low DIBIL ratio	Moderate DIBIL ratio	High DIBIL ratio		***p*** value
		n = 10,260	n = 3420	n = 3420		n = 3420	
**Angiography findings**							
	LM/three-vessel	6740 (65.7)	2146 (62.7)	2213 (64.7)		2381 (69.6)	<0.001
	Proximal LAD	2931 (28.6)	1009 (29.5)	969 (28.3)		953 (27.9)	0.304
	PCI/CABG	6212 (60.5)	2120 (62.0)	2047 (59.9)		2045 (59.8)	0.107

CABG: Coronary Artery Bypass Grafting, LM: left main vessel, LAD: left anterior descending artery, PCI: percutaneous coronary intervention

### DIBIL ratio predicted the occurrence of a poor prognosis

**Fig. 3:**
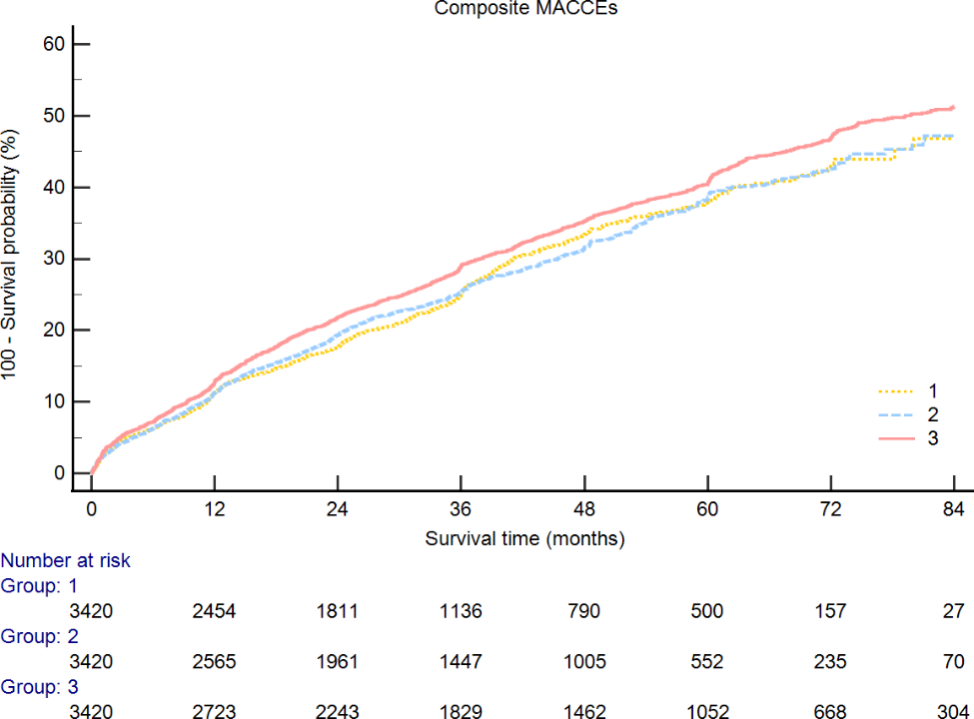
Kaplan-Meier curves for composite MACCEs. (MACCEs, major adverse cardiac and cerebral events; HR, hazard ratio; CI, confidence interval)

**Fig. 4:**
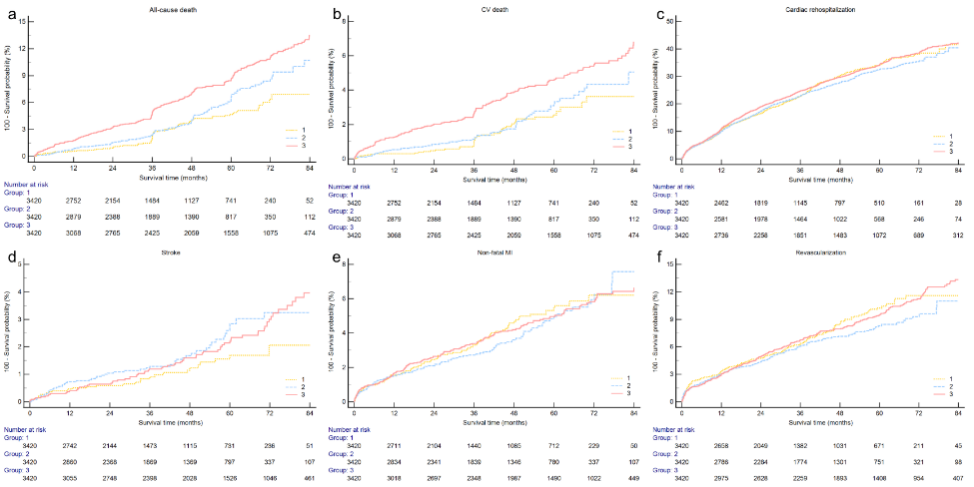
Kaplan-Meier curves for all-cause death(a), cardiac death (b), cardiac rehospitalization (c), stroke (d), non-fatal MI (e), revascularization (f) of the DIBIL ratio < 0.20 group (line 1), 0.20 ≤ DIBIL ratio < 0.26 group (line 2) and DIBIL ratio ≥ 0.26 group (line 3). (MI, myocardial infarction; DIBIL ratio, direct-indirect bilirubin ratio; HR, hazard ratio; CI, confidence interval)

During the follow-up period, the incidence of composite MACCEs is 2974 (29.0%) in the total enrolled population, in the low DIBIL ratio group the incidence is 810 (23.7%), and 902 (26.4%) in the moderate DIBIL ratio group, 1262 (36.9%) in high DIBIL ratio group (Table 3). The Kaplan-Meier curves show that the cumulative rate of composite MACCE (Fig. 3) was not statistically different between the three groups. But the high DIBIL ratio group had a significantly higher cumulative rate of all-cause death (Fig. 4a) and CV death (Fig. 4b). In addition, the cumulative rate is also shown no statistical difference in cardiac rehospitalization (Fig. 4c), stroke (Fig. 4d), non-fatal MI (Fig. 4e), and revascularization (Fig. 4f).

**Table 3:** Clinical outcomes

Variable	Total population n = 10,260	Low DIBIL ratio n = 3420	Moderate DIBIL ratio n = 3420	High DIBIL ratio n = 3420	***p*** value
All-cause death	498 (4.9)	87 (2.5)	131 (3.8)	280 (8.2)	<0.001
CV death	252 (2.5)	46 (1.3)	63 (1.8)	143 (4.2)	<0.001
Non-fatal MI	376 (3.7)	112 (3.3)	112 (3.3)	152 (4.4)	0.010
Cardiac rehospitalization	2507 (24.4)	724 (21.2)	770 (22.5)	1013 (29.6)	<0.001
Revascularization	710 (6.9)	216 (6.3)	204 (6.0)	290 (8.5)	<0.001
Stroke	159 (1.5)	32 (0.9)	55 (1.6)	72 (2.1)	<0.001
Composite MACCEs	2974 (29.0)	810 (23.7)	902 (26.4)	1262 (36.9)	<0.001

CV: cardiovascular, MACCEs: Major Adverse Cardiac and Cerebrovascular events, MI: myocardial infarction

In Table 4, the univariate and multivariate Cox regression analyses were employed to predict the incidence of all-cause death. According to the univariate analysis, the predictor linked to all-cause death occurrence were direct bilirubin, DIBIL ratio, age, BMI, systolic blood pressure, heart rate, hypertension history, diabetes history, previous stroke, previous MI, past PCI and CABG, β-blocker, and statin use, WBC, hemoglobin, hs-CRP, RBG at admission, FPG, HbA1c, albumin, ALT, ALP, ChE, creatinine, eGFR, TC, TGs, LVEF, LM or three-vessel involved, antiplatelet agents and statin use during hospitalization. FPG, RBG at admission, TGs, and HbA1c had a high correlation (p < 0.001). ALT, ALP and ChE also had a great correlation (p < 0.001). Creatinine was significantly correlated with eGFR (p < 0.001), meanwhile, hs-CRP was significantly correlated with WBC (p < 0.001). Therefore, FPG, RBG at admission, ALP, ChE, creatinine, and hs-CRP were not included in the final multivariate model. In the following multivariate Cox proportional hazards, regression analysis indicated that DIBIL ratio, age, BMI, systolic blood pressure, heart rate, previous stroke, hemoglobin, HbA1c, albumin, ALT, eGFR, LVEF, LM, or three-vessel involved independently predicted the incidence of all-cause death in patients with extremely high-risk of ASCVDs.

**Table 4:** Independent predictors of all-cause death

		Univariate		Multivariate	
		HR (95%CI)	***p*** value	Adjusted HR (95%CI)	***p*** value
Total bilirubin, umol/L		1.011 (0.997, 1.025)	0.132		
Direct bilirubin, umol/L		1.109 (1.079, 1.140)	<0.001	1.069 (1.035, 1.103)	< 0.001
Indirect bilirubin, umol/L		0.996 (0.976, 1.015)	0.650		
Direct/indirect bilirubin ratio		3.339 (2.352, 4.739)	<0.001	2.652 (1.577, 4.461)	<0.001
Age, years		1.088 (1.078, 1.099)	<0.001	1.056 (1.042, 1.070)	<0.001
Male gender		1.161 (0.967, 1.393)	0.110		
BMI, kg/m^2^		0.936 (0.911, 0.961)	<0.001	0.966 (0.938, 0.995)	0.023
SBP, mmHg		1.009 (1.004, 1.014)	<0.001	1.007 (1.001, 1.013 )	0.027
DBP, mmHg		0.987 (0.979, 0.995)	0.002	0.993 (0.982, 1.004)	0.225
Heart rate, bpm		1.024 (1.019, 1.030)	<0.001	1.014 (1.007, 1.020)	<0.001
**Medical history**					
	Current/ex-Smoker	1.031 (0.864, 1.231)	0.732		
	Hypertension	1.391 (1.132, 1.710)	0.002	1.047 (0.817, 1.342)	0.716
	Diabetes	1.412 (1.182, 1.686)	<0.001	0.878 (0.685, 1.125)	0.304
	Dyslipidemia	0.854 (0.714, 1.021)	0.084		
	Previous Stroke	2.170 (1.777, 2.649)	<0.001	1.427 (1.139, 1.788)	0.002
	Previous MI	1.970 (1.575, 2.464)	<0.001	1.215 (0.899, 1.641)	0.206
	Past PCI	1.380 (1.110, 1.716)	0.004	1.199 (0.900, 1.596)	0.215
	Past CABG	2.153 (1.404, 3.301)	<0.001	1.389 (0.842, 2.291)	0.199
**Medication on admission**					
	Antiplatelet agent	1.033 (0.863, 1.237)	0.722		
	ACEI/ARB	1.060 (0.882, 1.274)	0.536		
	Beta-blocker	0.783 (0.625, 0.980)	0.033	0.865 (0.663, 1.128)	0.284
	Statins	0.699 (0.568, 0.862)	0.001	0.789 (0.615, 1.013)	0.063
**Laboratory data**					
	WBC, 10^9^/L	1.047 (1.011, 1.084)	0.010	1.038 (0.995, 1.083)	0.085
	Hemoglobin, g/L	0.988 (0.985, 0.991)	<0.001	0.995 (0.990, 1.000)	0.032
	HsCRP, mg/L	1.033 (1.026, 1.041)	<0.001		
	RBG at admission, mmol/L	1.062 (1.041, 1.084)	<0.001		
	FPG, mmol/L	1.104 (1.071, 1.139)	<0.001		
	HbA1c, %	1.176 (1.111, 1.243)	<0.001	1.169 (1.085, 1.260)	<0.001
	Albumin, g/L	0.858 (0.839, 0.877)	<0.001	0.970 (0.943, 0.998)	0.034
	ALT, U/L	0.988 (0.982, 0.995)	0.001	0.990 (0.983, 0.998)	0.009
	AST, U/L	1.001 (1.001, 1.002)	0.086		
	ALP, U/L	1.008 (1.004, 1.011)	<0.001		
	GGT, U/L	1.002 (1.000, 1.004)	0.054		
	ChE,	0.685 (0.647, 0.725)	<0.001		
	Creatinine, umol/L	1.025 (1.021, 1.029)	<0.001		
	eGFR, ml/min/1.73m2	0.961 (0.957, 0.966)	<0.001	0.988 (0.981, 0.994)	<0.001
	TC, mmol/L	0.910 (0.833, 0.994)	0.037	0.976 (0.870, 1.094)	0.674
	TGs, mmol/L	0.720 (0.639, 0.811)	<0.001	0.924 (0.813, 1.050)	0.224
	LDLC, mmol/L	0.943 (0.837, 1.063)	0.337		
	HDLC, mmol/L	0.783 (0.558, 1.098)	0.156		
**Echocardiography**					
	LVEF	0.005 (0.003, 0.011)	<0.001	0.034 (0.013, 0.091)	<0.001
**Angiography findings**					
	LM/three-vessel	2.936 (2.284, 3.774)	<0.001	1.666 (1.248, 2.224)	0.001
	Proximal LAD	1.411 (1.172, 1.700)	<0.001	1.026 (0.832, 1.265)	0.813
	PCI/CABG	1.049 (0.876, 1.258)	0.601		
**Medication during hospitalization**					
	Antiplatelet agent	0.634 (0.405, 0.992)	0.046	0.684 (0.394, 1.188)	0.178
	ACEI/ARB	1.328 (1.105, 1.597)	0.003	0.995 (0.794, 1.245)	0.962
	Beta-blocker	1.009 (0.831, 1.225)	0.931		
	Statins	0.714 (0.537, 0.949)	0.020	0.837 (0.581, 1.205)	0.339

ACEI: angiotensin-converting enzyme inhibitors, ARB: angiotensin receptor blockers, ALT: alanine aminotransferase, AST: aspartate amino transferase, ALP: alkaline phosphatase, BMI: body mass index, CABG: Coronary Artery Bypass Grafting, CRP: c-reactive protein, ChE: Cholinesterase, DBP: diastolic blood pressure, eGFR: estimated glomerular filtration rate, FPG: fast plasma glucose, GGT: gamma glutamyl transpeptidase, HDL-C: high density lipoprotein cholesterol, LDL-C: low density lipoprotein cholesterol, LVEF: left ventricular ejection fraction, MI: myocardial infarction, NSTEMI: non-ST elevated myocardial infarction, PCI: percutaneous coronary intervention, RBG: random blood glucose, SBP: systolic blood pressure, STEMI:ST-elevated myocardial infarction, TC: total cholesterol, TGs: triacylglycerol, UAP: unstable angina pectoris, WBC: white blood cells

Table 5 presented the competing risk regression analysis for MACCEs. On unadjusted competing risk modeling, the cumulative incidence of all-cause death, CV death, and nonfatal stroke increased significantly with elevated DIBIL ratio levels (p < 0.05). Multivariate-adjusted hazard ratio (HR) also indicated that a high DIBIL ratio was correlated with a high incidence of all-cause death, CV death (p < 0.05).

**Table 5:** Competing risk model of clinical outcomes

		Unadjusted HR (95% CI)	***p*** value	Adjusted HR (95% CI)	***p*** value
All-cause death					
	DIBIL ratio<0.20	Ref		Ref	
	0.20 ≤ DIBIL ratio<0.26	1.343 (1.024, 1.761)	0.033	1.269 (0.954, 1.688)	0.102
	DIBIL ratio ≥ 0.26	2.220 (1.742, 2.829)	<0.001	1.829 (1.405, 2.381)	<0.001
CV death					
	DIBIL ratio<0.20	Ref		Ref	
	0.20 ≤ DIBIL ratio<0.26	1.202 (0.821, 1.760)	0.345	1.152 (0.772, 1.717)	0.489
	DIBIL ratio ≥ 0.26	1.966 (1.392, 2.776)	<0.001	1.600 (1.103, 2.321)	0.013
Non-fatal MI					
	DIBIL ratio<0.20	Ref		Ref	
	0.20 ≤ DIBIL ratio<0.26	0.904 (0.696, 1.175)	0.452	0.922 (0.700, 1.215)	0.565
	DIBIL ratio ≥ 0.26	0.955 (0.745, 1.225)	0.718	0.918 (0.689, 1.222)	0.556
Cardiac rehospitalization					
	DIBIL ratio<0.20	Ref		Ref	
	0.20 ≤ DIBIL ratio<0.26	0.958 (0.865, 1.060)	0.406	0.942 (0.847, 1.048)	0.272
	DIBIL ratio ≥ 0.26	1.040 (0.945, 1.146)	0.423	0.997 (0.896, 1.110)	0.959
Revascularization					
	DIBIL ratio<0.20	Ref		Ref	
	0.20 ≤ DIBIL ratio<0.26	0.831 (0.684, 1.011)	0.064	0.839 (0.683, 1.029)	0.092
	DIBIL ratio ≥ 0.26	0.985 (0.823, 1.178)	0.868	0.965 (0.791, 1.178)	0.725
Stroke					
	DIBIL ratio<0.20	Ref		Ref	
	0.20 ≤ DIBIL ratio<0.26	1.592 (1.025, 2.474)	0.039	1.378 (0.873, 2.175)	0.169
	DIBIL ratio ≥ 0.26	1.586 (1.043, 2.412)	0.031	1.189 (0.756, 1.870)	0.453
Composite MACCEs					
	DIBIL ratio<0.20	Ref		Ref	
	0.20 ≤ DIBIL ratio<0.26	0.959 (0.866, 1.062)	0.419	0.945 (0.850, 1.052)	0.303
	DIBIL ratio ≥ 0.26	0.991 (0.899, 1.092)	0.858	0.959 (0.862, 1.068)	0.451

CV: cardiovascular, DIBIL: direct/indirect bilirubin ratio, MACCEs: Major Adverse Cardiac and Cerebrovascular events, MI: myocardial infarction

### Enhancing the impact of DIBIL ratio on predictive value for all-cause death

**Fig. 5:**
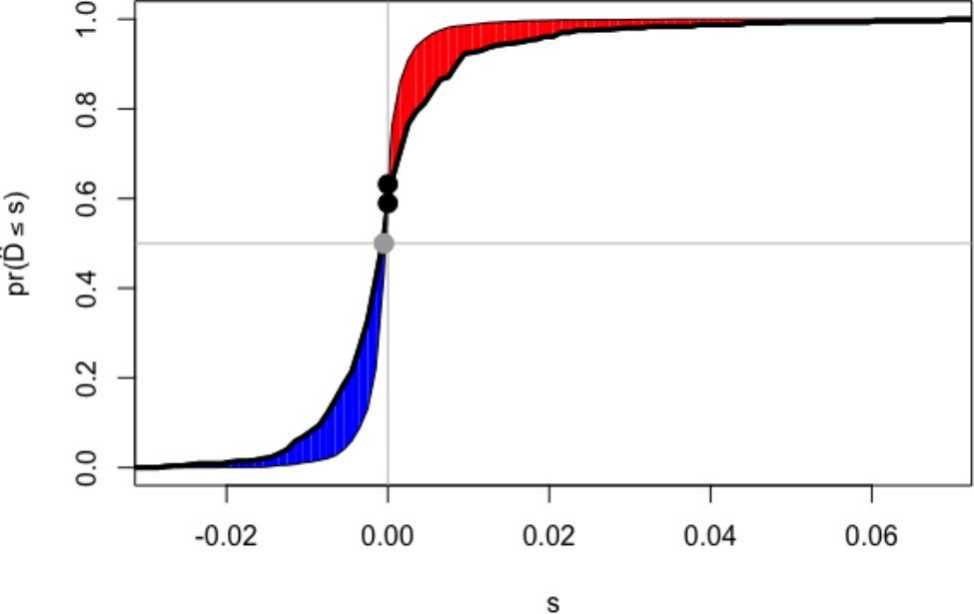
IDI of DIBIL ratio compared with Baseline risk model of all-cause death. Baseline risk model including age, BMI, SBP, heart rate, history of stroke, hemoglobin, albumin, HbA1c, ALT, eGFR, LVEF, LM/three vessels in angiography findings. (IDI, integrated discrimination improvement; DIBIL ratio, direct-indirect bilirubin ratio; BMI, body mass index; SBP, systolic blood pressure; HbA1c, glycated hemoglobin; ALT, alanine aminotransferase; eGFR, estimated glomerular filtration rate; LVEF, left ventricular ejection fraction; LM: left main vessel)

Table 6; Fig. 5 showed that compared with total bilirubin, DB, IDB, DIBIL ratio significantly improved the reclassification and discrimination ability beyond the baseline risk model with IDI 0.004(0, 0.010), p < 0.001; C-index 0.805(0.783–0.827), p < 0.001.

**Table 6:** Predictive value and predictive power of various models

	IDI			C-index		
	Index	95% CI	*p* value	Index	95% CI	*p* value
Baseline risk model				0.801	0.778, 0.823	<0.001
Total bilirubin	0.002	0, 0.004	0.040	0.802	0.808, 0.848	<0.001
Direct bilirubin	0.002	0, 0.004	<0.001	0.803	0.782, 0.828	<0.001
Indirect bilirubin	0.001	0, 0.002	0.182	0.801	0.776, 0.825	<0.001
Direct/Indirect bilirubin ratio	0.004	0, 0.010	<0.001	0.805	0.783, 0.827	<0.001

Baseline risk model including age, BMI, SBP, heart rate, history of stroke, hemoglobin, albumin, HbA1c ALT, eGFR, LVEF, LM/three vessels in angiography findings

ALT: alanine aminotransferase, BMI: body mass index, eGFR: estimated glomerular filtration rate, HbA1c: glycated hemoglobin, IDI, integrated discrimination improvement, SBP: systolic blood pressure, LVEF: left ventricular ejection fraction, LM: left main vessel

## Discussion

To our knowledge, this is the first study to explore the relationship between the DIBIL ratio and MACCEs in extremely high-risk ASCVD patients. The main findings of our study include: (1) The AUC of the DIBIL ratio is significantly higher than DBIL, TBIL, and IBIL. indicating that DIBIL ratio is a better biomarker for the prediction of all-cause death; (2) the incidences of MACCEs significantly increased with a higher DIBIL ratio; (3) The DIBIL ratio is an independent predictor of all-cause death; (4) The addition of DIBIL ratio to a baseline risk model had an enhancive impact on the predictive value for death. Conclusively, we confirmed that the DIBIL ratio was positively interrelated to increased poor prognosis.

ASCVD remained the leading cause of mortality in China, it’s extremely necessary to assign risk estimates to apply prevention strategies. Patients with extremely high-risk ASCVD usually suffered higher morbidity and mortality potential (30% or greater 10-year MACCEs risk) [16]. Therefore, more and more studies focused on figuring out potential biomarkers for better management of this population.

As the product of heme catabolism, bilirubin has been investigated as a biomarker for the prognosis of ASCVD. However, there are many controversies about this parameter. On the one hand, Yue et al. [17] reported that increased direct bilirubin was associated with more all-cause death in ACS patients. Chenbo and colleagues [12] also found that high TB and DB but not IDB was associated with a higher risk of MACCEs in Chinese ACS. This trend is consistent with our findings. While exploring the underlying mechanisms, Gupta et al. [9] reported that bilirubin could act as a scavenger of the reactive oxygen species independent of the conjugated or unconjugated forms. Additionally, bilirubin was reported to reduce arterial stiffness according to a preclinical test in diabetic mice [18]. Also, preclinical studies on mice demonstrated the protective effects of bilirubin on hypertension induced by angiotensin-II [19]. On the other hand, some studies found an inverse association between plasma bilirubin and total mortality. HAPIEE cohort [20] indicated that there was a negative correlation between bilirubin and mortality. In addition, other studies reported a U-shaped association between TBIL, IDB, and CHD risk. From the biological aspects, first, a high level of bilirubin is an indicator of oxidative stress and inflammation, which is a friend and foe to the pathological process of ASCVD. Second, a high level of bilirubin is an indicator of liver dysfunction, which may also cause cell apoptosis. From the clinical aspects, we found that this divergence may be due to several aspects, first, the study design and the definitions of the endpoints have a great impact on the results. Second, some studies elucidated the relationship between bilirubin and coronary artery diseases in random patients but not under acute stress conditions, such as ACS, which may cause antipodal conclusions. Currently, several studies performed to evaluate the relationship between bilirubin and acute coronary syndrome and found that the major adverse cardiac events were more frequent in the high bilirubin group [21]. This conclusion is consistent with our study. Third, when patients suffered ACS especially those comorbid with heart failure, there is usually evidence of liver dysfunction, such as the increased aspartate amino transferase and alanine aminotransferase [17], increased bilirubin could also reflect liver dysfunction, from this perspective, higher serum bilirubin could contribute to increased cardiac risk. Indirect bilirubin is metabolized and transferred into direct bilirubin in the liver, depending on liver function to a great extent. All the above papers analyzed the relationship between total, indirect or direct bilirubin and the endpoints, which may draw different even opposite conclusions. Considering this issue, to resolve the discrepancies, we first investigated the prognostic value of DIBIL ratio, total bilirubin, direct bilirubin, and indirect bilirubin in our enrolled patients, and found that the DIBIL ratio is the best indicator.

In this study, we evaluated the prognostic value of the DIBIL ratio in patients with extremely high-risk of ASCVD in different types of MACCEs and its subgroups and found that a higher DIBIL ratio was related to a higher incidence of all-cause death and CV death in competing risk model. Additionally, we also found that adding the DIBIL ratio to the baseline risk model had an enhancing impact on the predictive value for all-cause death. We held the idea that all the above findings may help physicians to predict the occurrence of clinical events and made relative strategies to prevent them. Another novelty of our study is that we identified that the DIBIL ratio was closely associated with all-cause death in different subgroups divided by age, BMI, systolic blood pressure, heart rate, previous stroke, hemoglobin, HbA1c, albumin, ALT, eGFR, LVEF, LM or three-vessel involved. Similar to previous studies, multiple linear regression indicated that factors including age, heart rate, diabetes, LM, or three-vessel involved related to total bilirubin [14]. ALT is a biomarker of liver function, increased ALT usually indicated liver dysfunction, in our study, we found that the DIBIL ratio is related to ALT and albumin after multiple regression analysis, this finding revealed that in ACS patients, especially those with extremely high-risk ASCVD, many patients also suffer liver dysfunction, which inferred that we should pay attention to the liver protection while we used bilirubin to predict patients’ prognosis. Published evidence has reported a negative association between bilirubin concentrations and metabolic syndrome and diabetes [22]. However, in our study, we found that a higher DIBIL ratio is positively related to HbA1c, this may be due to the patients included in the study, in our study, we enrolled patients with extremely high-risk ASCVD, while Lin’s work mainly focused on children and adolescents. More studies should be done to retest our conclusions in the future. Accordingly, compared with simple direct or indirect bilirubin, the DIBIL ratio may be a better marker for prognosis. Finally, although our data showed that the DIBIL ratio increased the discrimination ability beyond the baseline risk model with IDI 0.004(0, 0.010), p < 0.001, this improvement is not significant, one possible explanation of this may be due to the excellent ability of the baseline risk model.

There are several limitations of our study. First, this was a single-center study only collecting a sample from Beijing Friendship Hospital, thus, there is no evidence to generalize conclusions in our study to other organizations. Second, this is a retrospective observed study, in the future, more prospective studies even RCTs are required to confirm our findings. Third, some laboratory parameters in our study were only measured once during hospitalization, which could cause potential bias. In addition, the biological mechanisms linking bilirubin and ASCVD risk are still unclear, future studies in this field may be necessary.

## Conclusion

Conclusively, this study firstly demonstrated that an increased DIBIL ratio was an independent predictor of poor prognosis in patients diagnosed with ACS. Additionally, the DIBIL ratio along with the baseline risk model exerts an enhancing impact on the predictive value for all-cause death.

## Electronic supplementary material

Below is the link to the electronic supplementary material.

Supplementary Material 1: Extreme high-risk ASCVD

Supplementary Material 2: Z test of AUCs in Figure 2

## Data Availability

The datasets generated and/or analyzed during the current study are not publicly available due to the provisions of the CBD Bank but are available from the corresponding author on reasonable request.

## Abbreviations

ACS: acute coronary syndrome
ALP: Alkaline Phosphatase
ALT: alanine aminotransferase
AMI: acute myocardial infarction
ASCVD: atherosclerotic cardiovascular diseases
BMI: body mass index
CAD: Coronary artery disease
CABG: Coronary Artery Bypass Grafting
CBD: Center of Beijing Friendship Hospital Database
CRP: c-reactive protein
CV death: cardiac and cerebral death
DB: direct bilirubin
DIBIL ratio: direct-indirect bilirubin ratio
DBIL: direct bilirubin
TBIL: total bilirubin
IBIL: indirect bilirubin
DM: diabetes mellitus; eGFR:estimated glomerular filtration rate; FPG:fast plasma glucose; HbA1c:glycated hemoglobin; HDL-C:high-density lipoprotein cholesterol; IDB:indirect bilirubin
IDI: integrated discrimination improvement
IQR: interquartile range
LDL-C: low-density lipoprotein cholesterol
LM: left main vessel
LVEF: left ventricular ejection fraction
MACCEs: major adverse cardiac and cerebral events
NSTEMI: non-ST segment elevation myocardial infarction
OGTT: oral glucose tolerance test
PCI: percutaneous coronary intervention
RBG: random blood glucose
ROC: Receiver-operating characteristic
SBP: systolic blood pressure
SCORE: systematic coronary risk evaluation
PCE: Pooled Cohort Equations
SD: standard deviation
STEMI: ST segment elevation myocardial infarction
TC: total cholesterol
TG: triglyceride
WBC: white blood cells
