# Supplementary material for: The predictive effect of direct-indirect bilirubin ratio on clinical events in acute coronary syndrome: results from an observational cohort study in north China
Source: BMC Cardiovasc Disord. 2022 Nov 10;22:478. doi: 10.1186/s12872-022-02894-1 (PMC9650858; doi:10.1186/s12872-022-02894-1)
Supplement: Supplementary file 1 — Supplementary Material 1: Extreme high-risk ASCVD [file 12872_2022_2894_MOESM1_ESM.docx]

| **Major servere ASCVD events** |
| --- |
| Recent ACS (within the past 12 months) History of MI (more than 12 months) History of ischemic stroke Symptomatic peripheral arterial disease (history of claudication with ABI <0.85, or previous revascularization or amputation) |
| **High risk Conditions** |
| Diabetes mellitus Hypertension CKD (eGFR 15-59 mL/min/1.73 m^2^)  Current smoking  History of prior coronary artery bypass surgery or percutaneous coronary intervention outside of the major ASCVD event(s)  Heterozygous familial hypercholesterolemia  Persistently elevated LDL-C (LDL-C ≥100 mg/dL [≥2.6 mmol/L]) despite maximally tolerated statin therapy and ezetimibe History of congestive heart failure  History of premature clinical ASCVD (male<55 years of age, female <65 years of age)  Multi-vascular lesions |

**Extreme high-risk ASCVD**
